# Cationic and Anionic Antimicrobial Agents Co-Templated Mesostructured Silica Nanocomposites with a Spiky Nanotopology and Enhanced Biofilm Inhibition Performance

**DOI:** 10.1007/s40820-022-00826-4

**Published:** 2022-03-29

**Authors:** Yaping Song, Qiang Sun, Jiangqi Luo, Yueqi Kong, Bolin Pan, Jing Zhao, Yue Wang, Chengzhong Yu

**Affiliations:** 1 Australian Institute for Bioengineering and Nanotechnology, The University of Queensland, Brisbane, QLD 4072 Australia; 2 Centre for Microscopy and Microanalysis, University of Queensland, Brisbane, QLD 4072 Australia; 3 Australia Centre for Water and Environmental Biotechnology, University of Queensland, Brisbane, QLD 4072 Australia; 4 School of Chemistry and Molecular Engineering, East China Normal University, Shanghai, 200241 People’s Republic of China

**Keywords:** Antibacterial surfactants, Spiky nanoparticles, Antibacterial, Anti-biofilm, Mesostructured nanocomposites

## Abstract

**Highlights:**

- A ‘dual active templating’ strategy is firstly reported, using cationic and anionic bactericidal agents as co-templates for the preparation of antibacterial silica nanocomposite with spiky nanotopography.
- The spiky nanocomposite exhibited enhanced antibacterial and biofilm inhibition performance, compared to pure antimicrobial cationic agent templated smooth silica nanocomposite.

**Abstract:**

Silica-based materials are usually used as delivery systems for antibacterial applications. In rare cases, bactericidal cationic surfactant templated silica composites have been reported as antimicrobial agents. However, their antibacterial efficacy is limited due to limited control in content and structure. Herein, we report a “dual active templating” strategy in the design of nanostructured silica composites with intrinsic antibacterial performance. This strategy uses cationic and anionic structural directing agents as dual templates, both with active antibacterial property. The cationic-anionic dual active templating strategy further contributes to antibacterial nanocomposites with a spiky surface. With controllable release of dual active antibacterial agents, the spiky nanocomposite displays enhanced anti-microbial and anti-biofilm properties toward *Staphylococcus epidermidis*. These findings pave a new avenue toward the designed synthesis of novel antibacterial nanocomposites with improved performance for diverse antibacterial applications.
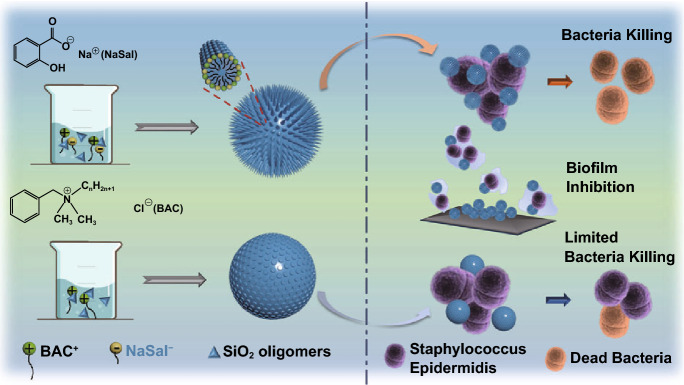

**Supplementary Information:**

The online version contains supplementary material available at 10.1007/s40820-022-00826-4.

## Introduction

Mesostructured materials such as mesoporous silica have attracted much attention during the past decades since their early reports in the 1990s [1–4]. Mesoporous silica nanoparticles (MSNs) have been applied in biocatalysis [5, 6], antitumor [7, 8] and specially antibacterial applications [9–12] due to unique properties including adjustable particle/pore sizes, high pore volume and excellent biocompatibility [13, 14]. MSNs generally act as carriers for the delivery of antibacterial compounds [15–20]. To create the mesopores, surfactants are removed for subsequent loading of drug molecules [21, 22]. Recently, a bactericidal reagent, benzalkonium chloride (BAC) was reported to act as the cationic surfactant to template the synthesis of mesostructured silica composite material. The antibacterial activity of BAC is due to the electronic interaction between cationic ammonium head group and negatively-charged bacterial membrane as well as the lipophilic tail enhanced membrane permeability, leading to bacterial membrane rupture and a leakage of cytoplasmic materials [23, 24]. However, the obtained composite particles have an large size of 650–850 nm, a small mesopore size of 18 Å, a low BAC release percentage of < 8% (in acidic condition) thus limited bactericidal efficiency (bacteria alive after 6 h treatment) [9, 25]. Therefore, it is highly desired to design novel silica based antibacterial nanocomposites with controlled structural properties and improved efficacy.

Among various strategies to combat bacterial infection [26, 27], creating spiky nanotopography has been reported with enhanced bacterial membrane adhesion and physical damage performance [28–30]. This strategy has also been applied to engineer antimicrobial nanoparticles with a rough surface topology and enhanced performance [31, 32]. For the synthesis of spiky silica nanoparticles, there are mainly two methods. One approach is using co-assembly of silica and polymer [9], while the other via surfactant cylindrical micelle templating in an oil/water biphasic system [33]. In these reports, extra treatment steps such as calcination or extraction are needed to remove the template to get the spiky surface, similar to most MSNs prepared as nanocarriers for further loading of antibacterial agents [34, 35]. To date, there are rare reports on the preparation of bactericidal surfactant containing silica nanocomposites with a spiky surface.

**Scheme 1:**
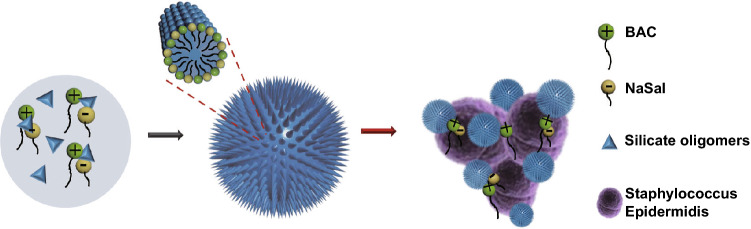
Schematic illustration for the synthesis, enhanced antibacterial and anti-biofilm activity of spiky nanocomposite **I** co-templated by BAC and NaSal

Herein, a “dual active templating” strategy is reported to synthesize bactericidal silica nanocomposites with a spiky surface, using cationic and anionic dual templates that are both active antibacterial agents. As shown in Scheme 1, BAC is used as a cationic structure-directing agent and sodium salicylate (NaSal) as an anionic agent. It is noted that sodium salicylate (NaSal) has antibacterial activity [36, 37], and has been utilized as a co-template to finely adjust the structures of MSNs (e.g., with large pores) [38, 39]. However, neither its content nor bactericidal property in silica nanocomposites has been reported. The use of BAC and NaSal as co-templates leads to a spiky silica nanocomposite **I.** Compared to nanocomposite **II** with a smooth surface templated by pure BAC, nanocomposite **I** showed high BAC loading (~ 22.0 wt%), efficient release (BAC and NaSal) (> 75% in 24 h), improved bactericidal activity and enhanced biofilm inhibition (70%) toward Gram-positive bacteria *Staphylacoccus Epidermidis* (*S. Epidermis*). The dual active templating strategy developed from this study may pave the way for the designed synthesis of novel functional nanocomposites for antibacterial applications.

## Experimental Section

### Materials and Reagents

Benzalkonium chloride (≥ 95%), triethanolamine (TEA, 99%), tetraethyl orthosilicate (TEOS, 98%), sodium salicylate (≥ 95%), hydrofluoric acid (HF reagent, 48%), hydrochloric acid (HCI, 37%), crystal violet solution (CV, 1% aqueous solution), phosphate buffer solution (PBS, 10 mM, pH = 7.4), and dead cell staining buffer propidium iodide (PI, Minimum Purities ≥ 95%) were purchased from Sigma-Aldrich. Green-fluorescent nucleic acid stains (SYTOTM 9, 5 mM) were purchased from Thermo Fisher Scientific. *S. Epidermis* (American Type Culture Collection (ATCC)-12,228) was purchased from ATCC.

### Synthesis of Nanocomposite I and II

Nanocomposites **I** with a spiky morphology containing BAC/NaSal/silica were synthesized using BAC and NaSal as co-templates and TEOS as the silica source. In a typical synthesis, 68 mg of TEA was added into 25 mL of deionized water and stirred at 80 °C for 30 min. Then, around 0.7 mL of 50% aqueous BAC and 80 mg of NaSal were added into the above solution and stirred for 1 h at 80 °C (molar ratio: NaSal/BAC = 0.5). After addition of 3 mL of TEOS, the solution was further stirred at 80 °C for another 2 h. Final nanocomposite I was collected by centrifugation at 25,200 RCF for 5 min, washing with ethanol for three times, and vacuum dried at 50 °C for 12 h. Part of nanocomposite **I** was calcined at 550 °C under air for 5 h and denoted as **I**-calcined. Nanocomposite **II** with a smooth surface was prepared vis a similar method, using only BAC as the template and TEOS as the silica precursor. Specifically, 68 mg of TEA was added into 25 mL of deionized water and stirred at 80 °C for 30 min. Then, 0.7 mL of 50% aqueous BAC was added into the above solution and stirred for 1 h at 80 °C. After the addition of 3 mL of TEOS, the solution was further stirred at 80 °C for another 2 h. The final nanocomposite **II** was collected by centrifugation at 25,200 RCF for 5 min, washing with ethanol for three times, and vacuum dried at 50 °C overnight.

### Quantification of BAC and NaSal Contents in Nanocomposites

Ultraviolet–visible spectroscopy (UV–Vis) analysis was applied to determine the contents of BAC and NaSal in silica nanocomposite. Nanocomposites were dissolved in HF at room temperature for 4 h and then diluted for UV–Vis analysis. The NaSal and BAC contents were determined and calculated at 299 and 209 nm, respectively (see details in Results and Discussion section).

### Characterization

Transmission electron microscopy (TEM) study was performed using J HT7700-EXALENS with an accelerated voltage of 80–100 kV. Scanning electron microscope (SEM) measurements were conducted using a JEOL JSM 7800 field-emission scanning electron microscope (FE-SEM). Energy-dispersive X-ray (EDX) mapping analysis was carried out using Hitachi HF5000 Cs-STEM/TEM. Dynamic light scattering (DLS) measurement was conducted at 25 °C using the Zetasizer Nano-ZS from Malvern Instruments. Before measurements, the samples were dispersed in deionized water by ultra-sonication, and all samples were measured for three times. Attenuated total reflectance Fourier transform infrared spectroscopy (ATR-FTIR) analysis was conducted on a ThermoNicolet Nexus 6700 FTIR spectrometer equipped with Diamond ATR Crystal. Nitrogen adsorption–desorption analysis was measured by a Micromeritcs Tristar II system at 77 K. Before the measurement, samples were degassed at 353 K overnight on a vacuum line. The total pore volume was calculated from the adsorbed amount at the maximum relative pressure (P/P_0_) of 0.99. The pore size of samples was calculated through Barrrett-Joyner-Halenda (BJH) method from the adsorption branches of the isotherms. The Brunauer–Emmett–Teller (BET) method was used to calculate the specific surface areas. Cross-Polarization Magic Angle Spinning ^13^Carbon Nuclear Magnetic Resonance (^13^C CP/MAS NMR) spectrum was measured by a solid-state Bruker Avance III spectrometer with 7 T (300 MHz for 1H) magnet, Zirconia rotor, 4 mm, rotated at 7 kHz. ICP-OES was performed to provide the quantitative measurement of silica adhered or uptaken by bacterial after 4 h incubation. A Thermo Scientific iCAP 6500 ICP-OES instrument was used, and the analysis was duplicated. The bacterial suspension and nanocomposite solution were mixed at the same ratio and condition as the antibacterial test. After 4 h culturing, the solution was filtered through 450 nm-pore filter membrane and washed with PBS twice. Then, the filter paper was dissolved in 10% HF solution for 24 h before the ICP-OES quantification of silicon content. The silicon amount was calculated based on each bacteria, and the bacterial number was determined by optical density (OD) reading at 600 nm.

### Drug Release Study

The release study was investigated by dispersing nanocomposite in pH 7.4 or pH 5 PBS solutions shaking at 37 °C at 220 RPM. Released BAC and NaSal were determined by analyzing the collected supernatant at different time points using UV–Vis.

### Antibacterial Activity

The antimicrobial capability of nanocomposite **I** was tested in *S.epidermidis* using Luria–Bertani (LB) -agar plates assay. Nanocomposite **II**, BAC, NaSal, calcined nanocomposite **I** and mixture of BAC/NaSal were selected as control. The nanocomposites were sterilized by dissolved in 70% (v/v) ethanol, followed by washing with sterilized PBS for three times before bacterial culture based on a reported protocol [40]. All the tests were conducted under acidic LB medium (pH = 5) with the tested BAC concentrations of 1, 2, 4 μg mL^−1^. The mixture of bacteria suspension (1.0 × 10^7^ CFU mL^−1^), acidic LB medium and nanocomposites/drug was incubated in 37 °C shaker at 220 RPM for 24 h and examined by LB-agar plate assay. 200 μL of treated bacterial suspensions were spread on sterilized LB-agar plates. After incubation at 37 °C for overnight, photographs were taken, and the bacteria colonies grown in each plate were counted.

### In vitro Biofilm Inhibition

For biofilm inhibition study, 200 μL of *S. Epidermidis* bacterial suspension (1.0 × 10^8^ CFU mL^−1^) added with BAC or nanocomposite **I** at 4 μg mL^−1^ BAC was added to 24-well plates and cultured at 37 ℃ without shaking. After 12 h incubation, the supernatant was removed. The samples were washed with 85% NaCl aqueous solution three times and then stained by SYTO9 and PI for confocal microscopy. Another plate was prepared for CV staining. The untreated biofilm was denoted as the control, and all experiments were performed in duplicates.

### Biocompatibility Assay

Cell viability was evaluated in Human Embryonic Kidney (HEK239T) cells using 3-(4,5-dimethylthiazol-2-yl)-2,5-diphenyl tetrazolium bromide (MTT) assay. HEK cells were seeded in a 96-well flat-bottom plate with a density of 7000–8000 cells per well for 24 h. Then, cell culture medium was replaced with fresh medium containing PBS, nanocomposite **I**/**II** and calcined nanocomposite **I** at a BAC concentration of 1 μg mL^−1^. After incubation in the incubator at 37 ℃ for 24 h, 20 μL of MTT solution (5 mg mL^−1^) was added to each well and cells were incubated for another 4 h. Then medium was replaced with 100 μL of dimethyl sulfoxide (DMSO). Then absorbance readings were measured at the wavelength of 570 nm using a microplate reader. The cells incubated with PBS were used as the control. All experiments were performed four times.

## Results and Discussion

**Fig. 1:**
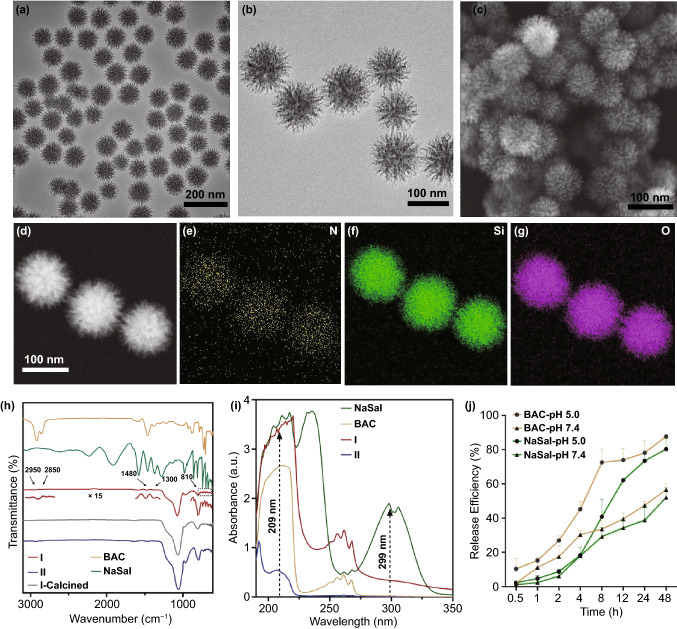
**a**, **b** TEM, **c** SEM and **d**–**g** EDX mapping images of nanocomposite **I**. **h** FTIR spectra of nanocomposites **I** and **II**, calcined nanocomposite **I**, BAC and NaSal. **I**. **i** UV–Vis spectra of nanocomposites **I** and **II.** **j** Time-dependent release profile of BAC and NaSal from nanocomposite **I** at pH 7.4 and 5.0

The nanocomposite **I** was prepared in an aqueous system using TEOS as the silica precursor, BAC as the cationic surfactant and NaSal as an anionic co-templating agent [38]. Low and high magnification TEM images (Fig. 1a, b) showed that nanocomposite **I** possesseds a spiky structure and a uniform particle size of 100 ± 10 nm by measuring 50 particles. The spiky surface morphology was further visualized in the SEM image (Fig. 1c**)**. Uniform distribution of nitrogen, silicon and oxygen elements in nanocomposite **I** was revealed by the energy-dispersive X-ray spectroscopy (EDX)-mapping results (Fig. 1d–g). The observation of nitrogen indicates the existence of BAC in the nanocomposite **I**. However, the presence of NaSal cannot be confirmed from these results.

To understand the role of NaSal in the synthesis and antibacterial application, the nanocomposite **II** was fabricated via a similar synthetic protocol without the addition of NaSal. Nanocomposite **II** exhibited a smooth surface and similar diameter to Nanocomposite **I** as shown in TEM (Fig. S2a, b) and SEM images (Fig. S2c). EDX mapping results of nanocomposite **II** also showed uniform distribution of nitrogen, silicon and oxygen elements (Fig. S2d–g). The relatively weak nitrogen signal indicated the content of BAC in nanocomposite **II** was lower than that in nanocomposite **I**. FTIR was further conducted to characterize the existence of BAC and NaSal. Compared to the spectrum of BAC (Fig. 1h), nanocomposites **I** and **II** exhibited typical peaks at 2950, 2850, and 1480 cm^−1^ corresponding to C–H stretching bands and benzene ring originated from BAC [23], suggesting the existence of BAC, which is consistent with EDX mapping results. The peaks originated from NaSal at 1300, 810, and 620–690 cm^−1^ are only observed from nanocomposite **I** [38]. The typical peaks of BAC and NaSal were not observed in **I**-calcined, suggesting complete removal of surfactants after calcination. ^13^C MAS NMR was conducted to characterize the compositions in nanocomposite **I**. As shown in Fig. S3, The typical peaks at 134 and 137 ppm were originated from benzyl chain of NaSal [41]. The peaks at 51 and 57 ppm are assigned to N(CH_3_)_2_ and NCH_2_ species, respectively; and the peak around 29 ppm is attributed to hydrophobic carbon chain in BAC [42]. These observations indicate the presence of BAC and NaSal in nanocomposite **I**.

The nitrogen sorption analysis was conducted to characterize the porous structure. The adsorption–desorption isotherms of nanocomposite **I** and nanocomposite **II** are shown in Fig. S2h. The major capillary condensation steps of nanocomposite **I** occurred at two high relative pressure (P/P_0_) steps, one around 0.90 and the other at > 0.97. The first capillary condensation step corresponds to a broad pore size distribution (Fig. S2h-inserted) centered at 26.3 nm for nanocomposite **I**, presumably reflecting the mean pore size of the spiky layer. The second capillary condensation step at higher P/P_0_ of 0.97 is attributed to packing voids between particles [33], which is also observed in the adsorption–desorption isotherm of nanocomposite **II**. The physical properties of nanocomposites **I** and **II** are summarized in Table S1. Compared to BAC templated nanocomposite **II** with a specific surface area of 86 m^2^ g^–1^ and a pore volume of 0.34 cm^3^ g^−1^, nanocomposite **I** templated by BAC/NaSal showed a higher specific surface area of 290 m^2^ g^–1^ and a pore volume of 0.69 cm^3^ g^−1^. Considering their difference in morphology (Figs. 1a–c and S2–c), the higher specific surface area of nanocomposite **I** than nanocomposite **II** is mainly attributed to the nanostructured spiky rough surface, which contains a portion of mesopores as evidenced in Fig. S2h. The zeta potential of three particles was measured (Table S1). Compared to nanocomposite **II**, nanocomposite **I** exhibited a higher surface charge, in accordance with a higher BAC content in nanocomposite **I** which is beneficial for antibacterial performance. After calcination, calcined-**I** exhibited the negative surface charge, indicating the successful removal of BAC in the nanocomposite.

Time-dependent TEM images of intermediated structures at different timepoints were collected to investigate the formation mechanism of nanocomposite **I** (Fig. S4). At the reaction time of 15 min, nanospheres with an average diameter of ~ 100 ± 5 nm were observed (Fig. S4a). A few rod-like structures were found deposited on the nanosphere outer surface. With the reaction time prolonged to 20 and 40 min, the nanoparticles with clear spike structure were observed as shown in Fig. S4b-c. Considering that in the absence of NaSal nanocomposite **II** a smooth surface formed, it is proposed that the interaction between negatively charged Sal^–^ and positively charged BAC^+^ decreases the charge density and thus the hydrophilic head group area in the BAC^+^/Sal^–^/silicate assembly, leading to an increase in packing parameter (g) and structural transition from spherical composite micelles toward cylindrical structures [38]. As reported by Zhao and co-workers, the epitaxial growth of cylindrical structures contributes to the growth of spiky silica nanocomposite **I** [33].

To quantitatively measure BAC and NaSal contents in the nanocomposites, a protocol based on UV–Vis spectroscopy was developed. As shown in Fig. 1i, the UV–Vis absorption spectrum of pure NaSal showed a peak at 299 nm, while at this wavelength pure BAC or nanocomposite **II** prepared in the absence of NaSal showed negligible absorbance. Therefore, the content of NaSal in nanocomposite **I** was determined from the standard curve obtained at 299 nm, which was calculated to be 4.28 wt% (Fig. S2j, top). To quantify the BAC content, the standard curves of NaSal and BAC at 209 nm were measured (Fig. S2j middle and bottom). By deducting the absorbance contributed from NaSal, the BAC content in nanocomposite **I** calculated was determined to be 21.96 wt%. Similarly, the percentage of BAC in nanocomposite **II** was determined to be 14.92 wt%, lower than that in nanocomposite **I**. Thermogravimetric analysis (TGA) was further conducted to determine the surfactants loading contents of nanocomposites. The weight loss before 110 °C is attributed to the removal of moisture. The weight loss in the temperature range between 110 and 600 °C should be attributed to the decomposition of templates and silica condensation, which was calculated to be 37.2% for nanocomposite **I** (mainly BAC and NaSal) and 20.3% (mainly BAC) for nanocomposite **II**. The difference between nanocomposites **I** and **II** is similar to that obtained from UV–Vis analysis. The higher loading contents analyzed from TGA compared to UV–Vis can be attributed to the further condensation of silanol groups.

Furthermore, the release of BAC and NaSal from nanocomposite **I** as a function of time was studied by UV–Vis spectroscopy (Fig. 1j**)**. The release test was conducted in PBS at two pH values (7.4 or 5), considering the acidic pH at the bacterial infection site [43]. Compared to the relatively slower release of BAC and NaSal (< 40% within 12 h) at neutral pH, nanocomposite **I** exhibited a higher release percentage of BAC/NaSal (e.g., >  ~ 60% at 12 h). For BAC release at pH 5, a burst release stage before 12 h and a subsequent sustained release stage (> 12 h) were observed. Moreover, the BAC release percentage at 24 h from nanocomposite **I** (~ 45% at pH 7.4; ~ 70% at pH 5) was significantly higher than that from nanocomposite **II** (< 20% at pH 7.4; ~ 36% at pH 5). The dissolved silicon content was also measured by ICP-OES. After 24 h, the concentration of dissolved silicon under pH 5 (23.3 mg L^−1^) was higher than that at pH 7.4 (10.8 mg L^−1^). Therefore, the increased release percentage of BAC at acidic pH is presumably due to the faster silica degradation as well as the replacement of benzalkonium ions by H^+^ under more acidic conditions [20]. The higher BAC release percentage of nanocomposite **I** compared to nanocomposite **II** is probably due to the faster degradation rate of large pore sized structure, which is consistent with a literature report where large-pore sized MSNs exhibited faster degradation than MSNs with smaller pore sizes [44]. The increased release of active molecules in nanocomposite **I** with spiky surface is beneficial for antibacterial applications.

**Fig. 2:**
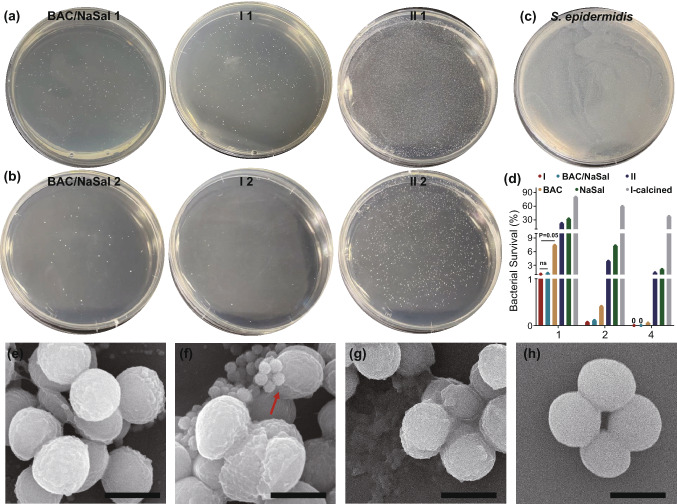
**a**, **b** Dose-dependent killing of S. epidermidis by BAC/NaSal, nanocomposites **I** and **II**. Photographs of plates containing treated culture, where 1/2 represents the BAC concentration of each group. **c** Photograph of plate containing tenfold serial dilutions of untreated culture. **d** the average survival percentage of *S. epidermidis* upon exposure to each group quantified based on 1, 2 and 4.μg BAC mL^−1^ Statistical significance is calculated using a two-tailed t test with significant p-values shown. “ns” denotes “not significant.” **e**–**h** SEM images of BAC/NaSal, nanocomposite **I**, nanocomposite **II** treated bacterial and untreated S.Epidermidis. (red arrows refer to a semi-spherical dent on bacterial surface upon adhesion of nanocomposite **I**; scale bar: 1 μm)

To demonstrate the advantage of nanocomposite **I** prepared by the “dual active templating” approach, its antibacterial activity toward *S. epidermis* was evaluated using plate counting method. The bacteria were cultured in acidic LB medium for 24 h, using nanocomposite **II**, **I**-calcined (see detailed characterization in Fig. S6), BAC/NaSal, BAC and NaSal as control groups due to more efficient release of BAC/Sal at acidic pH tested above. Nanocomposite **I**, BAC/NaSal, nanocomposite **II** (Fig. 2a, b), BAC or NaSal **(**Fig. S6**)** all showed dose-dependent antimicrobial activity compared to untreated group (Fig. 2c) while no obvious antibacterial activity was observed for calcined **I** (Fig. S6), suggesting the antibacterial function from silica is minimal. Less bacterial colony grown in BAC/NaSal treated group compared to either BAC or NaSal treated group, suggesting the enhanced antibacterial efficiency of the combination of BAC and NaSal. Least colony was observed for nanocomposite **I** group at all dosages compared to nanocomposite **II** or drug controls, suggesting the advantage of the spiky topography and BAC/NaSal compositions (Fig. 2d).

Compared to the bacterial viability of BAC and NaSal treated groups (7.39% and 32.27%, respectively), the survival rate of BAC/NaSal treated group was less (1.25%), implying the synergistic effect of the dual antibacterial agents in bacterial killing. Similar synergistic effect can also be evidenced by the higher survival rate of nanocomposite **II** treated group (7.59%) than nanocomposite **I** (1.07%) treated bacteria. The cell viability was also evaluated in Human Embryonic Kidney (HEK239T) cells. Compared to the 41% cell viability of pure drug treated group (BAC/Sal), nanocomposite **I**, **II** and calcined nanocomposite **I** all exhibited ~ 90% cell viability, suggesting excellent biocompatibility of the silica-based nano-formulations.

Next, SEM was conducted to visualize the morphology change in bacteria with or without nanocomposite treatment. Compared to smooth and intact membrane in untreated group (Fig. 2h), clear bacterial cell membrane damage with nanocomposite sinking into bacteria was observed in nanocomposite **I** treated group, creating some dents on bacterial surface (Fig. 2f red arrow), while limited membrane damage was observed in BAC/NaSal (Fig. 2e) or nanocomposite **II** (Fig. 2g) treated bacteria. These results collectively demonstrated that rough nanocomposite **I** templated by "dual actives" displayed the most effective bactericidal capability, presumably due to bacterial membrane disruption through spiky surface enhanced adhesion and boosted release of dual antibacterial agents (BAC and NaSal). The silicon content was analyzed by ICP-OES as shown in Fig. S8. Nearly 2 pg of nanocomposite **I** adhered on/uptaken by each bacteria, which was almost twofold of the silicon content of nanocomposite **II** or **I**-calcined treated bacteria. The results are consistent with the observation from SEM images. From Figs. 2d and S6, BAC/NaSal and nanocomposites **I** treated groups showed 100% bactericidal performance at the concentration of 4 μg BAC mL^−1^, thus 4 μg mL^−1^ was chosen for the following biofilm inhibition study.

**Fig. 3:**
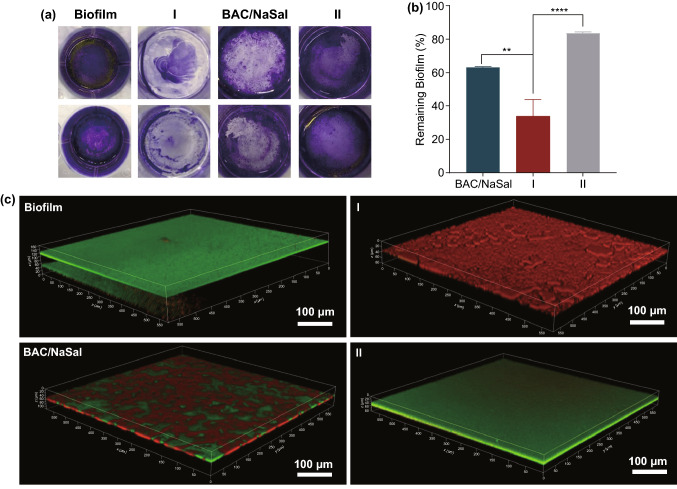
**a** Digital images of biofilms stained by CV. **b** Quantitative analysis of CV stained biofilm. **c** 3-D Confocal images of 24 h old biofilm treated by PBS, nanocomposite **I**, BAC/NaSal or nanocomposite **II**

The advantage of nanocomposite **I** over nanocomposite **II** and the BAC/NaSal group was further evaluated in their biofilm inhibition performance through examining their efficiency in inhibiting bacterial surface adhesion and biofilm formation. Nanocomposites or BAC/NaSal were added to planktonic bacteria at the concentration of 4 μg BAC mL^−1^. Crystal violet (CV) staining was applied to visualize remaining biofilm treated with or without nanocomposite [45], where the positively charged dye interacted with the negatively-charged cell wall and resulted in the purple color of CV [46]. As shown from the standard CV colorimetric assay (Fig. 3a), untreated control or nanocomposite **II** remained dark purple, suggesting more biofilm biomass, the BAC/NaSal or nanocomposite **I** treated groups exhibited significantly lighter color, suggesting its ability in biofilm inhibition. Quantification of biofilm biomass normalized to untreated group was shown in Fig. 3b, the inhibiting effect of nanocomposite **I** was significantly better (biofilm formation reduced to 33%) than nanocomposite **II (**85%) and BAC/NaSal (63%).

To better analyze the biofilm thickness, 3D confocal microscopy was conducted and LIVE/DEAD bacteria was stained and analyzed (Fig. 3c). The thickness of untreated biofilm was estimated to be 25 μm. Much thinner biofilms (less than 10 μm) with significantly increased dead cell populations (> 90%) were observed after 24 h incubation withnanocomposite **I** (Fig. 3c, I). For comparison, thicker biofilm was formed on BAC/NaSal (~ 15 μm) or nanocomposite **II** (22 μm biofilm) treated groups. The above results demonstrate that "dual actives templated" nanocomposite **I** shows excellent synergy in inhibiting biofilm formation.

## Conclusions

In summary, we have reported a “dual active templating” strategy and successfully synthesized spiky silica nanocomposite containing two active agents for antibacterial applications. The dual active templating strategy contributes to not only the formation of a spiky surface that enhances bacterial membrane adhesion and physical damage, but also an effective release of two bactericidal components for synergistically improved bacterial killing and biofilm inhibition. These findings provide a new strategy for the designed synthesis of novel functional silica composite materials with boosted performance in antibacterial applications.

## Supplementary Information

Below is the link to the electronic supplementary material.

Supplementary file1 (PDF 880 KB)
