# Supplementary material for: Cationic and Anionic Antimicrobial Agents Co-Templated Mesostructured Silica Nanocomposites with a Spiky Nanotopology and Enhanced Biofilm Inhibition Performance
Source: Nanomicro Lett. 2022 Mar 29;14:83. doi: 10.1007/s40820-022-00826-4 (PMC8964905; doi:10.1007/s40820-022-00826-4)
Supplement: Supplementary file 1 — Supplementary file1 (PDF 880 KB) [file 40820_2022_826_MOESM1_ESM.pdf]

Supporting Information for

Cationic and Anionic Antimicrobial Agents Co-templated Mesostructured Silica Nanocomposites with a Spiky Nanotopology and Enhanced Biofilm Inhibition Performance

Yaping Song[1], Qiang Sun[2], Jiangqi Luo[1], Yueqi Kong[1], Bolin Pan[1], Jing Zhao[3], Yue Wang[1,] *, and Chengzhong Yu[1, 4, *]

[1] Australian Institute for Bioengineering and Nanotechnology, The University of Queensland, Brisbane, QLD 4072, Australia

[2] Centre for Microscopy and Microanalysis, University of Queensland, Brisbane, QLD 4072, Australia

[3] Australia Centre for Water and Environmental biotechnology, University of Queensland, Brisbane, QLD 4072, Australia

[4] School of Chemistry and Molecular Engineering, East China Normal University, Shanghai 200241, P. R. China

*Corresponding authors. E-mail: yue.wang1@uq.edu.au (Yue Wang), c.yu@uq.edu.au (Chengzhong Yu)

Supplementary Figures and Table

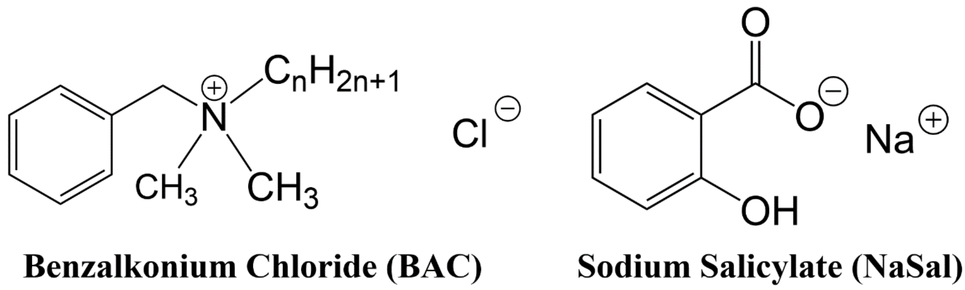

Fig. S1 Chemical structure of BAC and NaSal

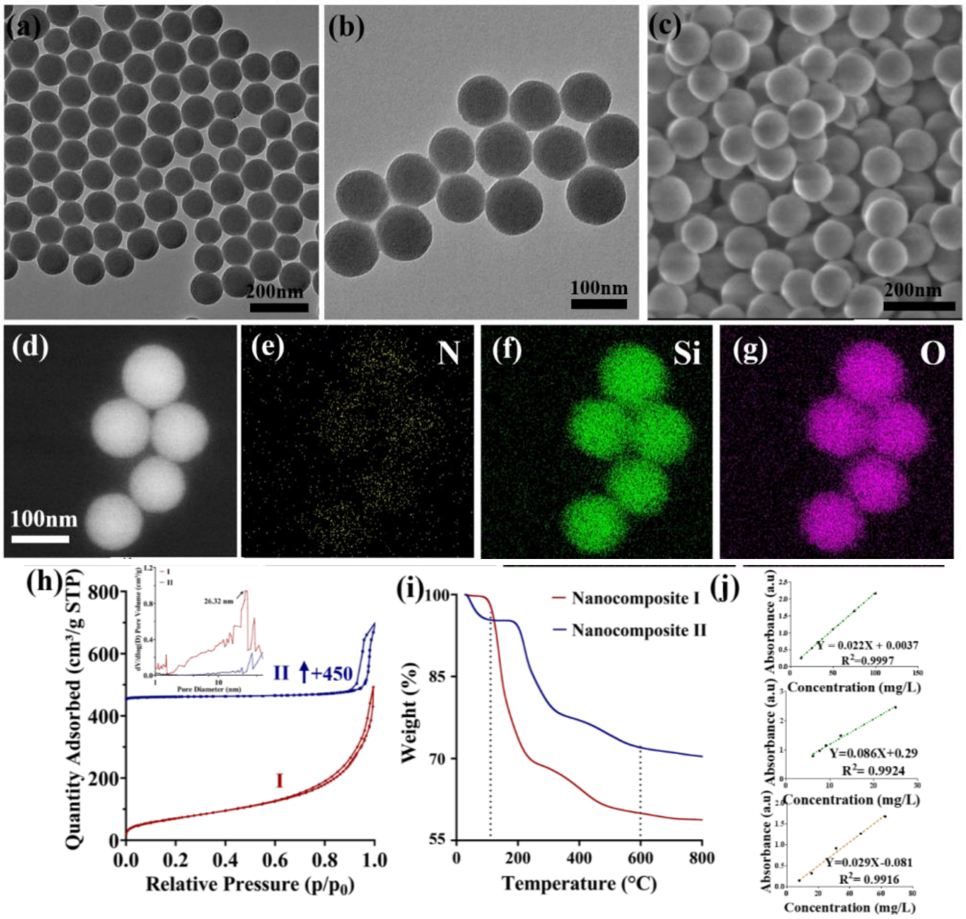

Fig. S2 TEM (**a, b**), SEM (**c**) and EDX elemental mapping (**d-g**) images of nanocomposite **II** Nitrogen adsorption-desorption isotherm (**h**) and (**h-inserted**) pore size distribution curves calculated from the adsorption branch using the Barrett–Joyner–Halenda (BJH) model of nanocomposite **I** and **II** TGA curves (**i**) of nanocomposite **I** and **II** Standard curves (**j**) for UV-Vis quantification of NaSal at 299 nm (top); NaSal at 209 nm (middle); and BAC at 209 nm (bottom)

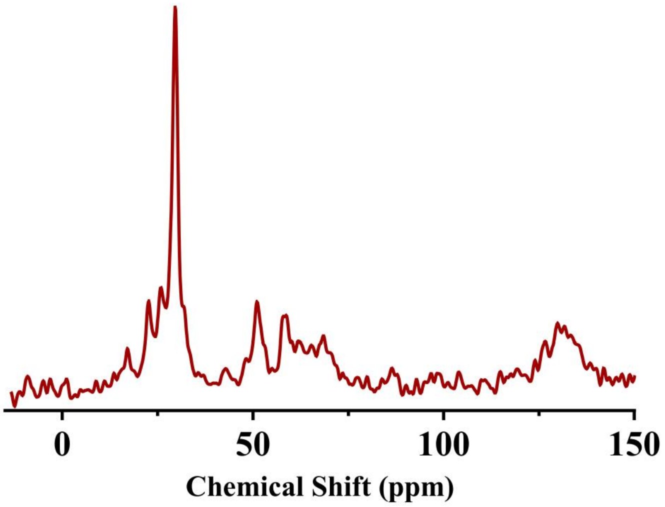

Fig. S3 ^{13}C MAS NMR spectrum of nanocomposite **I**

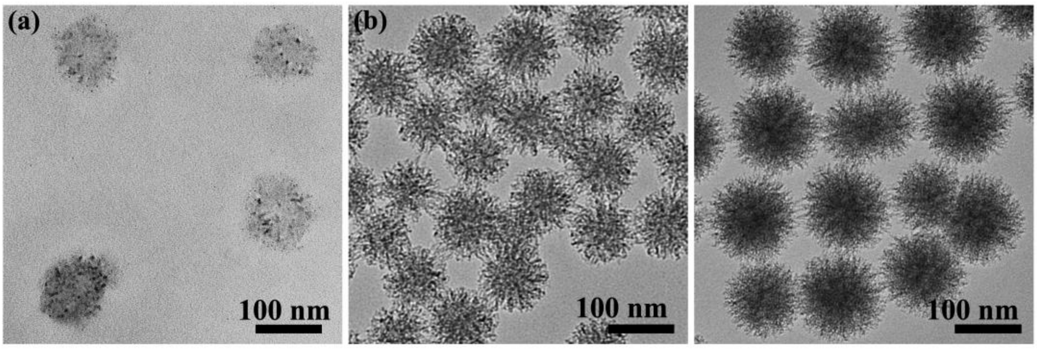

Fig S4 Time-dependent investigation of nanocomposite **I** TEM images of intermediate structures collected at reaction time of (**a**) 15 min, (**b**) 20 min and (**c**) 40 min

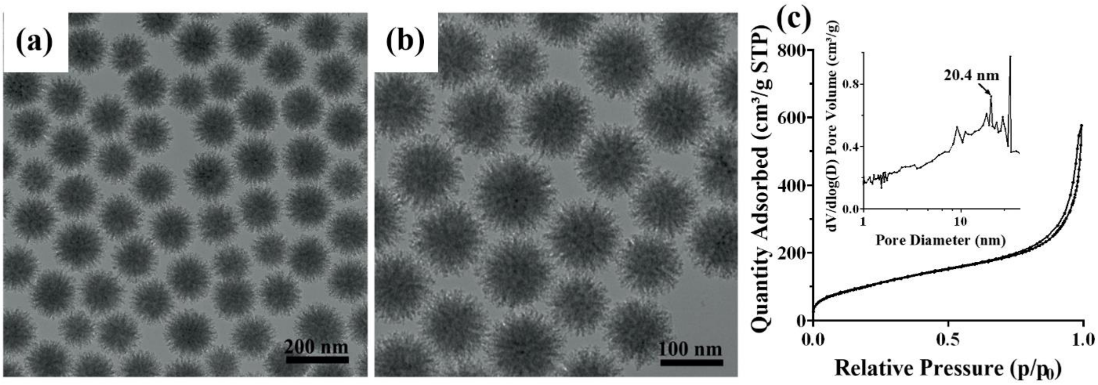

Fig S5 TEM images (**a**, **b**); Nitrogen adsorption-desorption isotherm (**c**) and pore size distribution curves (c-inserted) of calcined nanocomposite **I** (denoted as I-calcined). **I**-calcined was prepared through 550 °C calcination of nanocomposite **I**

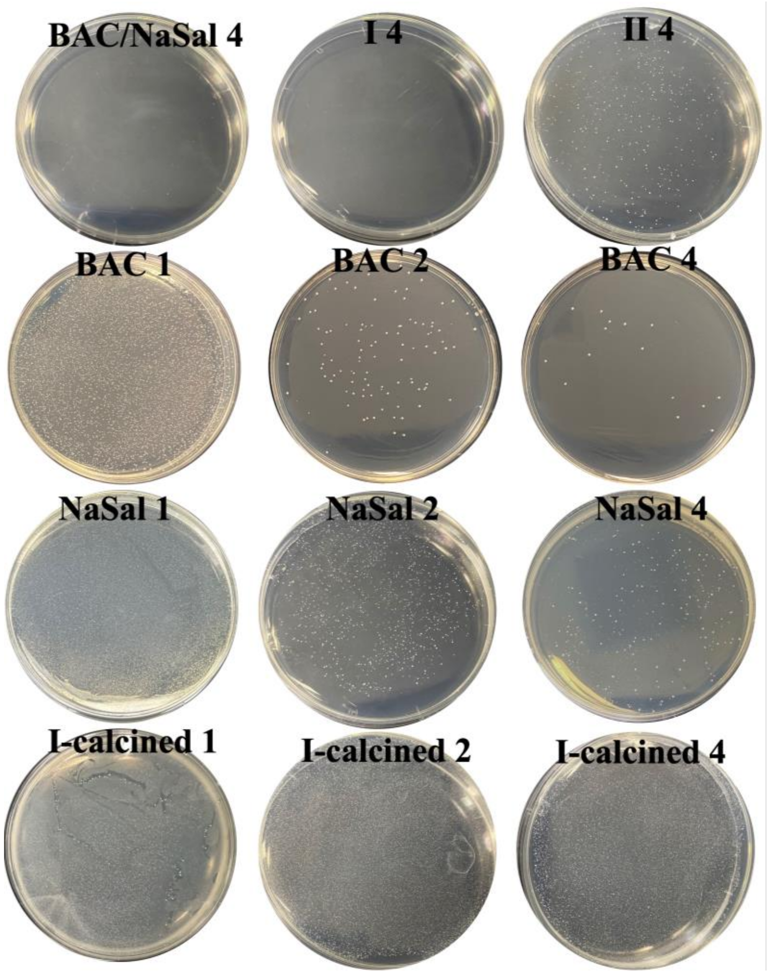

Fig. S6 Photographs of plates containing S. epidermidis culture treated with BAC, NaSal, and I-calcined (1, 2, and 4 represents the BAC concentration of 1, 2, and 4 μg L^{-1})

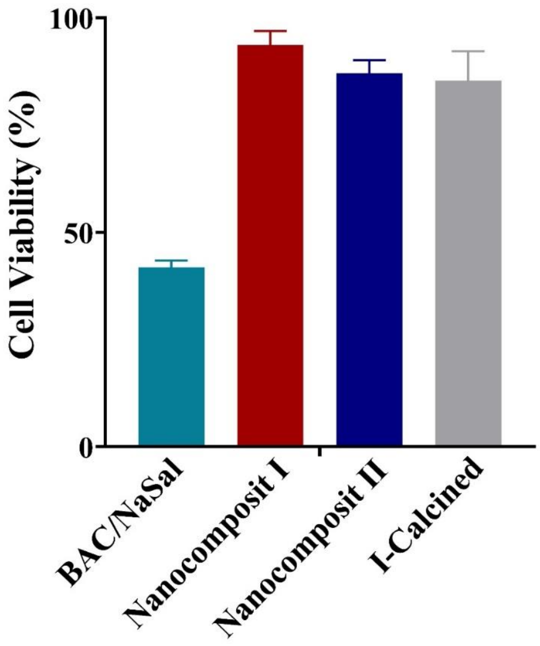

Fig. S7 Cytotoxicity in HEK239T cells after 24 h incubation with BAC/NaSal, nanocomposites **I**/**II**/ and calcined-**I** at the concentration of 1 μg BAC mL^{-1}

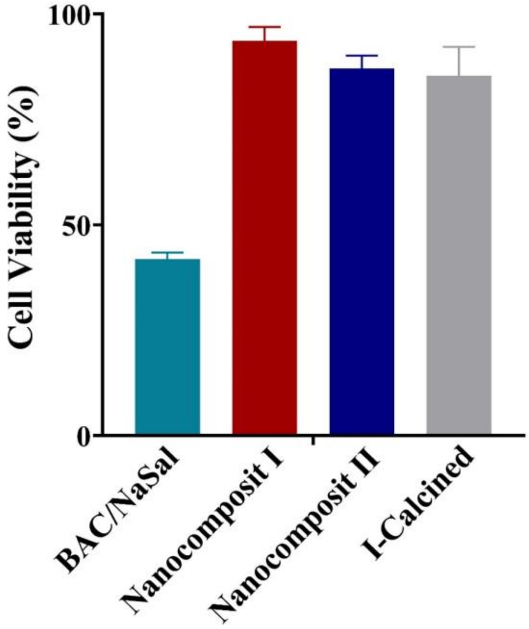

Fig. S8 Bacterial silica uptake/adhesion of nanocomposites **I**/**II** and **I**-Calcined at the concentration of 1 μg BAC mL^{-1}

Table S1 Physical Properties of nanoparticles

Sample name	Z-average (nm) /PDI	Surface area (m^2 g^{-1})	Pore volume (cm^3 g^{-1})	Surface charge (mV)
**Nanocomposite I**	139/0.10	290	0.69	-12.90
**Nanocomposite II**	114/0.18	86	0.34	-15.80
**I-calcined**	145/0.12	821	0.96	-23.55
